# Notes and News

**Published:** 1906-03-24

**Authors:** 


### NOTES AND NEWS.

It having been reported to the Westminster Guardians that a female patient who had been in the workhouse infirmary, a Belgian, was found to be in possession of over £100 in notes and gold, as well as a diamond ring valued at £60, they decided to deduct £8 for her keep, handing her back the remainder.

At the annual meeting of the General Board of the National Physical Laboratory last week, Lord Rayleigh in the chair, it was stated that the receipts for test work almost entirely on instruments had increased from £3,804 to £4,358, but that the receipts for special investigation had fallen. The new buildings are making rapid progress, and will probably be opened during the spring.

The Prince of Wales and the Chapter-General of the Order of the Hospital of St. John of Jerusalem in England recently awarded a bronze medal to Mr. Robert Manuel, a porter in the employ of the Cape Government Railway, for his gallantry in saving the life of an old man who had fallen on to the rails and was in imminent danger of being cut to pieces by an approaching train. On the 11th of last month the Duke of Connaught presented this medal to the owner, the occasion being a review of the Transvaal volunteers at Johannesburg.

The Governor's report of the Straits Settlement, recently issued, speaks briefly but satisfactorily of the condition and progress of the hospitals in this Colony. The majority are under Government management, while the estate hospitals, maintained by the planters, are under Government supervision. The total number of in-patients (exclusive of the lunatic asylum) treated in 1904 was 23,462. The present site of the Tan Tock Seng Hospital having been found unsuitable, the Government acquired a new site on rising ground near Balestier Road, and a sum of $100,000 for the new buildings was voted in the Budget services for 1905.

An epidemic of enteric fever, believed to be due to articles of bedding, is reported on board the training ship *Cornwall,* moored off Purfleet, and used for the reception of boys from reformatory schools. The sick boys, nineteen in number, have all been isolated.

At a drawing-room meeting on behalf of Mrs. Gladstone's Free Convalescent Home last week, Lord Ripon presiding, it was resolved to provide a ward of eight beds at the Home at Mitcham for surgical convalescents, and that the public be asked to support the scheme. The additional capital outlay, it was stated, would be about £150, and the cost of the maintenance of each bed would be greater than in the ordinary wards. But it was estimated that from 8s. to 10s. per week could be obtained from the hospitals for each surgical case admitted.

The Home Secretary received a deputation on Monday from several organisations, who urged the need of amending the present provisions of the Factory Act with regard to laundries. Mr. Gladstone, having listened to several speakers, said that he considered that laundries are the weak spot in factory and workshop law. He thought that the long hours of standing work in hot rooms, especially on the later days of the week, must be trying and injurious to women and young persons. He trusted that the Government would be able next session to bring in a Bill dealing with the matter.

The second reading of the Poisons and Pharmacy Bill was moved by the Earl of Crewe in the House of Lords on Tuesday evening. The Lord President of the Council, who said that the measure carries into effect the recommendations of the Departmental Committee on the sale of poisonous substances and as to what substances should be treated as poisons, disclaimed, on behalf of the Government, any desire to interfere unduly with companies carrying on chemists' businesses. He also expressed his readiness to consult the Pharmaceutical Society before the Committee stage of the Bill was reached. The second reading was agreed to.

Speaking at the annual meeting of the National Association for the Prevention of Consumption, on Tuesday, Sir William Broadbent observed that one of the great objects of the Association had been to stir up the local authorities to take steps to protect working people, who were chiefly concerned about consumption, as they were protected from other infectious diseases. The memorandum which had been issued by the Scottish Local Government Board at Edinburgh was a step in the right direction, and one which he hoped to see followed by the Local Government Board, at whose head was a man who know the conditions of the people and the necessity for such a step. Sir William went on to say, "It was somewhat of a temptation to think that there were 2,000 beds—reserved for small-pox—standing idle in the possession of the Asylums Board. These beds could be well utilised for the treatment of consumptive patients, but the Asylums Board was purely a Poor-law authority."

